# Association of Racial Discrimination With Adiposity in Children and Adolescents

**DOI:** 10.1001/jamanetworkopen.2023.22839

**Published:** 2023-07-11

**Authors:** Adolfo G. Cuevas, Danielle M. Krobath, Brennan Rhodes-Bratton, Shu Xu, Jesulagbarami J. Omolade, Aniyah R. Perry, Natalie Slopen

**Affiliations:** 1 Department of Social and Behavioral Sciences, New York University School of Global Public Health, New York; 2 Center for Anti-Racism, Social Justice, and Public Health, New York University School of Global Public Health, New York; 3 Friedman School of Nutrition Science and Policy, Tufts University, Boston, Massachusetts; 4 Eliot-Pearson Department of Child Study and Human Development, Tufts University, Medford, Massachusetts; 5 Department of Biostatistics, New York University School of Global Public Health, New York; 6 Department of Community Health, Tufts University, Medford, Massachusetts; 7 Department of Social and Behavioral Sciences, Harvard T.H. Chan School of Public Health, Boston, Massachusetts

## Abstract

**Importance:**

Childhood obesity is a major public health issue and is disproportionately prevalent among children from minority racial and ethnic groups. Personally mediated racism (commonly referred to as racial discrimination) is a known stressor that has been linked to higher body mass index (BMI; calculated as weight in kilograms divided by height in meters squared) in adults, but little is known about the association of racial discrimination and childhood and adolescent adiposity.

**Objective:**

To assess the prospective association between self-reported experiences of racial discrimination and adiposity (BMI and waist circumference) in a large sample of children and adolescents in the Adolescent Brain Cognitive Development (ABCD) study.

**Design, Setting, and Participants:**

This cohort study used complete data from the ABCD study (2017 to 2019), involving a total of 6463 participants. The ABCD study recruited a diverse sample of youths from across the US, with rural, urban, and mountain regions. Data were analyzed from January 12 to May 17, 2023.

**Exposure:**

The child-reported Perceived Discrimination Scale was used to quantify racial discrimination, reflecting participants’ perceptions of being treated unfairly by others or unaccepted by society based on their race or ethnicity.

**Main Outcomes and Measures:**

Weight, height, and waist circumference were measured by trained research assistants. BMI *z* scores were computed by applying the US Centers for Disease Control and Prevention’s age and sex-specific reference standards for children and adolescents. Waist circumference (inches) was quantified as the mean of 3 consecutive measures. Measurements were taken from time 1 (ie, 2017 to 2019) and time 2 (ie, 2018 to 2020).

**Results:**

Of the 6463 respondents with complete data, 3090 (47.8%) were female, and the mean (SD) age was 9.95 (0.62) years. Greater racial discrimination exposure at time 1 was associated with higher BMI *z *score in both unadjusted (β, 0.05; 95% CI, 0.02-0.08) and adjusted regression models (β, 0.04; 95% CI, 0.01-0.08). Discrimination at time 1 was associated with higher waist circumference in unadjusted (β, 0.35; 95% CI, 0.15-0.54) and adjusted (β, 0.24; 95% CI, 0.04-0.44) models.

**Conclusions and Relevance:**

In this cohort study of children and adolescents, racial discrimination was positively associated with adiposity, quantified by BMI *z *score and waist circumference. Interventions to reduce exposure to racial discrimination in early life may help reduce the risk of excess weight gain across throughout life.

## Key Points

**Question:**

Are self-reported experiences of racial discrimination associated with adiposity in a nationally representative sample of children and adolescents?

**Findings:**

In this cohort study of 6463 participants, racial discrimination at baseline was associated with higher adiposity (body mass index and waist circumference) 1 year later in unadjusted and adjusted regression models.

**Meaning:**

These findings suggest that personally mediated racial discrimination may be a risk factor for developing obesity in children and adolescents, above and beyond socioeconomic status.

## Teaser

This cohort study assesses the association of self-reported experiences of racial discrimination with adiposity in a large sample of children and adolescents in the US.

## Introduction

Obesity in children and adolescents is a critical public health issue in the US. Nearly 1 in 5 US children and adolescents have obesity, as indicated by a body mass index (BMI, calculated as weight in kilograms divided by height in meters squared) equal to or greater than the 95th percentile for their age and sex.^1^ This prevalence is greater than a 3-fold increase from the 5% prevalence in 1975.^1,2^ In addition, it is estimated that youth BMI trajectories have nearly doubled since the onset of the COVID-19 pandemic among children aged 6 to 11 years, and children who had obesity prepandemic were the worst impacted.^3^ Childhood and adolescent obesity confer a wide range of short- and long-term health consequences, including hypertension, hyperlipidemia, diabetes, sleep apnea, depression, and early mortality.^4,5^ Importantly, obesity prevalence is not equal across racial or ethnic groups in childhood or adulthood. Black and Hispanic children and adolescents have a higher prevalence of obesity compared with their non-Hispanic White and Asian or Asian American counterparts, placing Black and Hispanic children at an increased risk for obesity-related chronic diseases.^6,7^ Despite extensive research on some factors contributing to the increasing prevalence of obesity and obesity disparities, including parent education, single-parent households, poverty, and neighborhood,^8,9,10,11,12^ our understanding of other potential risk factors remains insufficient. This knowledge gap limits the development of effective interventions to reduce obesity and address existing inequities.

Personally mediated racism—here, referred to as racial discrimination— includes discriminatory actions, attitudes, or prejudices exhibited by individuals toward others based on their perceived racial or ethnic background.^13^ Exposure to racial discrimination has been recognized as a social determinant of health and a driver of health inequities among children and adolescents.^14,15,16^ Taunting, harassment, and other types of indignities can have a similar impact on the body as other psychological stressors. They can induce negative emotional reactions and, in turn, stimulate biological and behavioral responses that increase the risk of disease.^17,18,19^ Growing research documents that exposure to discrimination is associated with a range of health outcomes, including depression, sleep difficulty, health-compromising behaviors, and high cortisol levels in children and adolescents.^20,21^ Despite this, the connection between discrimination and BMI is not well understood. In studies with adults, where there are more studies available, some find a positive association,^22,23,24,25^ whereas others find no association.^26,27,28^ Research on youth has mostly relied on nonrepresentative samples. A study on a community-based sample of 198 African American youths found a positive association between racial discrimination and BMI.^29^ Additionally, in a smaller sample of Australian children (N = 124), researchers found that higher levels of reported discrimination were associated with increased cardiometabolic risk factors, including higher BMI, waist circumference, blood pressure, and inflammation.^19^ However, it is crucial to investigate the connection between racial discrimination and BMI in a large population-based sample of US children to comprehend the influence of racial discrimination on obesity rates among US children and adolescents.

This cohort study aims to quantify the prospective association between racial discrimination and adiposity (BMI and waist circumference) in a large and racially diverse sample of 9-to-11-year-olds. We hypothesized that higher levels of racial discrimination at time 1 (ie, 2017 to 2019) would be associated with a higher BMI and higher waist circumference at time 2 (ie, 2018 to 2020).

## Methods

This study used 2 waves of data from the Adolescent Brain Cognitive Development (ABCD) study, which recruited a diverse sample of youths from 21 sites across the US, including rural, urban, and mountain regions. The study’s primary goal was to examine brain development and the association of various individual, social, and familial contextual variables on health, well-being, and health-related behaviors.^30^ To ensure adequate racial or ethnic representation, the ABCD investigators used probability sampling methods in both public and private elementary schools. The analysis primarily examined data from the year 1 wave (2017 to 2019) as the baseline, which encompassed the measurement of racial discrimination. The year 2 wave (2018 to 2020) was used as the follow-up period. The sample comprised approximately 11 200 youths, with 51.1% boys and 48.9% girls. Additionally, 45.2% of the sample consisted of children from minoritized racial and ethnic groups, including 19.2% Latino or Hispanic, 16.0% Black, and 5.5% Asian or Pacific Islander children. Detailed information regarding recruitment methods and sampling strategies is provided elsewhere.^31^ Institutional review board approval was obtained from the University of California, San Diego, and each of the 21 study sites.^31^ Written informed consent was obtained from all participants prior to data collection and analysis. The study followed the Strengthening the Reporting of Observational Studies in Epidemiology (STROBE) reporting guideline to ensure transparency and completeness in reporting of methods and results.

### Measures

#### BMI

Trained research assistants measured weight and height. We calculated BMI using the US Centers for Disease Control and Prevention’s age and sex-specific reference categories in children and adolescents, and calculated *z* scores.^32^

#### Waist Circumference

Trained research assistants measured waist circumference. We calculated waist circumference as the mean of 3 consecutive measures in inches.

#### Racial Discrimination

We used the Perceived Discrimination (PD) Scale to assess interpersonal racial discrimination (eAppendix in Supplement 1).^33^ The scale contains 3 items asking respondents about the frequency they were treated unfairly or negatively because of their ethnic background by peers, teachers, and other adults; and 4 items about their feeling unaccepted in society because of their race or ethnicity. The scale uses 5 frequency response codes (0 = almost never, 1 = seldom, 2 = sometimes, 3 = often, 4 = very often). Consistent with prior studies,^33,34,35^ the scores were averaged, with higher scores indicating greater reports of racial discrimination.

#### Covariates

To address potential confounding factors, we used a primary regression model that included variables known to have independent associations with both discrimination exposure and obesity, as demonstrated in previous studies^20,36,37,38,39,40^: child’s age (years), parent-reported biological sex (female and male), household income (less than $25 000, $25 000-$50 000, $50 000-$75 000, $75 000-$100 000, $100 000-$200 000, and greater than $200 000), parent education (high school diploma or less, some college, a Bachelor’s or Associate’s degree, and a graduate or professional degree), and nativity status (US-born and foreign-born). In addition, we incorporated the adiposity measures from year 1 into the model to strengthen the investigation of the association between discrimination and adiposity at time 2. Our study aims to provide a direct measurement of racial discrimination exposure, and as such, it is essential that our results are valid and free from bias.^41,42,43,44^ Including parent-reported race or ethnicity in the model would compromise this validity, as it introduces the potential for social experiences and biases to impact our results. Race or ethnicity, particularly parent-reported race or ethnicity, is a complex social construct that reflects a person’s experiences and exposure to social advantages and oppression.^41,45^ Including this construct in our model could introduce bias and potentially confound our results, particularly as it relates to the impact of racial discrimination on BMI.

### Statistical Analysis

Descriptive analyses were performed to characterize the sample’s overall sociodemographic characteristics, racial discrimination exposure, and adiposity (BMI and waist circumference). Various correlation coefficients (Pearson, point-biserial, Spearman, Phi, and Rank biserial) were used to evaluate participant characteristic interrelations. Multiple linear regression models were used to quantify associations between racial discrimination and adiposity (BMI *z *score and waist circumference). A prospective analysis regressed time 2 BMI on time 1 reports of discrimination accounting for time 1 BMI. The same series of analyses were modeled using waist circumference as the primary independent variable. We tested the associations between racial discrimination and adiposity with and without adjusting for covariates. In this study, complete case analysis was used to address missing data in the multivariate models. This resulted in 5412 cases being omitted from the analysis due to the presence of missing values for any of the variables under examination. Statistical significance was set at *P* <.05, and all tests were 2-sided. All analyses were conducted from January 13 to May 17, 2023, using Stata SE version 17 (StataCorp).

## Results

### Descriptive Statistics

Of the 6463 respondents with complete data, 146 (2.3%) as Asian or Native Hawaiian or Pacific Islander, 679 (10.5%) identified as Black, 1173 (18.1%) as Hispanic or Latino of any race, 19 (0.3%) as Native American or American Indian, 3860 (59.7%) as White, 553 (8.6%) as multiracial, and 33 (0.5%) as other race, which includes all respondents who selected only the other race checkbox (Table 1). The sample included 3090 females (47.8%), and the mean (SD) age was 9.95 (0.62) years. Across the sample at time 1, 4015 (62.1%) of children were a healthy weight, while 458 (7.1%) were underweight, 1259 (19.5%) were overweight, and 731 (11.3%) had obesity. The mean (SD) overall racial discrimination score was 0.2 (0.4). Children with obesity reported the highest mean (SD) overall racial discrimination scores (0.21 [0.40]). Meanwhile, children with healthy weight reported a mean (SD) racial discrimination score of 0.15 (0.35). Most children (2104 [32.5%]) came from households with total incomes between $100 000 and $199 999. There was a significant difference in the prevalence of obesity according to household income categories. Obesity was most prevalent among children in the lowest income category (177 [24.1%]) and was least prevalent in the highest earning households (28 [3.6%]); χ^2^ = 355.16; *P* < .001). Children whose parents had a graduate degree had the lowest prevalence of obesity (106 [5.8%]) while those whose parents had a high school diploma or less had the highest obesity prevalence (22.4%; χ^2^ = 231.40; *P* < .001).

**Table 1.** Descriptive Statistics of the Adolescent Brain and Cognitive Development Study Sample, Overall and by Weight Category

Characteristic	Participants, No. (%) (N = 6463)					
	Overall	T1 Underweight	T1 Healthy weight	T1 Overweight	T1 Obesity	*P* value^a^
Participants	6463	458 (7.09)	4015 (62.12)	1259 (19.48)	731 (11.31)	NA
T1 BMI *z *score^b^						
Mean (SD)	0.42 (1.14)	−1.88 (0.60)	0.04 (0.63)	1.46 (0.24)	2.20 (0.25)	<.001
Median (IQR)	0.44 (−0.36 to 1.28)	−1.71 (−2.09 to −1.44)	0.08 (−0.44 to 0.55)	1.45 (1.25 to 1.65)	2.16 (2.01 to 2.35)	NA
T2 BMI *z *score^b^						
Mean (SD)	0.44 (1.14)	−1.47 (0.81)	0.07 (0.76)	1.39 (0.48)	2.09 (0.46)	<.001
Median (IQR)	0.47 (−0.34 to 1.31)	−1.49 (−1.88 to −1.02)	0.10 (−0.44 to 0.62)	1.44 (1.15 to 1.71)	2.13 (1.94 to 2.36)	NA
T1 waist circumference, in^b^	27.64 (4.63)	23.26 (2.50)	25.74 (2.79)	30.45 (2.76)	35.88 (4.22)	<.001
T2 waist circumference, in^b^	28.67 (4.91)	24.41 (2.34)	26.69 (2.85)	31.58 (3.42)	37.25 (4.89)	<.001
Discrimination score^b^	0.16 (0.36)	0.12 (0.29)	0.15 (0.35)	0.19 (0.39)	0.21 (0.40)	<.001
Median (IQR)	0 (0 to 0.14)	0 (0 to 0.14)	0 (0 to 0.14)	0 (0 to 0.28)	0 (0 to 0.28)	NA
Age, y^b^	9.95 (0.62)	10.02 (0.63)	9.96 (0.62)	9.94 (0.62)	9.9 (0.62)	.09
Parent-reported race or ethnicity^c^						
American Indian or Alaska Native	19 (0.29)	0	8 (42.11)	7 (36.84)	4 (21.05)	<.001
Asian, Native Hawaiian, or Pacific Islander	146 (2.26)	15 (10.27)	95 (65.07)	23 (15.75)	13 (8.90)	<.001
Black	679 (10.51)	23 (3.39)	335 (49.34)	156 (22.97)	165 (24.3)	<.001
Hispanic to any race(s)	1173 (18.15)	50 (4.26)	579 (49.36)	331 (28.22)	213 (18.16)	<.001
Multiracial, Non-Hispanic	553 (8.56)	47 (8.50)	333 (60.22)	102 (18.44)	71 (12.84)	<.001
White	3860 (59.72)	321 (8.32)	2645 (68.52)	636 (16.48)	258 (6.68)	<.001
Other^d^	33 (0.51)	2 (6.06)	20 (60.61)	4 (12.12)	7 (21.21)	<.001
Biological sex^c^						
Female	3090 (47.81)	254 (8.22)	1896 (61.36)	595 (19.26)	345 (11.17)	.009
Male	3373 (52.19)	204 (6.05)	2119 (62.82)	664 (19.69)	386 (11.44)	.009
Nativity status^c^						
Non-US born	180 (2.79)	8 (4.44)	117 (65.00)	29 (16.11)	16 (14.44)	.19
US born	6283 (97.21)	450 (7.16)	3898 (62.04)	1230 (19.58)	705 (11.22)	.19
Total household income to $^c^						
24 999 and below	735 (11.37)	24 (3.27)	345 (46.94)	189 (25.71)	177 (24.08)	<.001
25 000-49 999	910 (14.08)	52 (5.71)	490 (53.85)	206 (22.64)	162 (17.80)	<.001
50 000-74 999	919 (14.22)	67 (7.29)	554 (60.28)	185 (20.13)	113 (12.30)	<.001
75 000-99 999	1008 (15.60)	66 (6.55)	620 (61.51)	223 (22.12)	99 (9.82)	<.001
100 000-199 999	2104 (32.55)	170 (8.08)	1437 (68.30)	345 (16.40)	152 (7.22)	<.001
200 000 and above	787 (12.18)	79 (10.04)	569 (72.30)	111 (14.10)	28 (3.56)	<.001
Parent education level^c^						
High school or less	791 (12.24)	33 (4.17)	381 (48.17)	200 (25.28)	177 (22.38)	<.001
Some college	981 (15.18)	64 (6.52)	554 (56.47)	214 (21.81)	149 (15.19)	<.001
Associate’s or Bachelor’s	2873 (44.45)	222 (7.73)	1815 (63.17)	537 (18.69)	299 (10.41)	<.001
Graduate degree	1818 (28.13)	139 (7.65)	1265 (69.58)	308 (16.94)	106 (5.83)	<.001

Abbreviations: T1, time 1 (ie, 2017 to 2019); T2, time 2 (ie, 2018-2020).

^a^ *P* values determined using 1 way analysis of variance test for continuous variables and χ^2^ tests for categorical variables.

^b^ Continuous variables presented as mean (SD).

^c^ Categorical variables presented as No. (column %) in column 1 and as N (row %) in columns 2-5.

^d^ Includes all respondents who selected only the other race checkbox.

### Racial Discrimination and Adiposity

In the unadjusted model, greater racial discrimination exposure at time 1 was associated with a higher BMI at time 2 (β, 0.05; 95% CI, 0.02-0.08) (Table 2). After adjusting for covariates, racial discrimination exposure remained positively associated with BMI (β, 0.04; 95% CI, 0.01-0.08). In the unadjusted model, greater racial discrimination exposure at time 1 was associated with a higher waist circumference at time 2 (β, 0.35; 95% CI, 0.15-0.54) (Table 2). After adjusting for covariates, racial discrimination exposure remained positively associated with waist circumference (β, 0.24; 95% CI, 0.04-0.44).

**Table 2.** Results of Unadjusted and Adjusted Multiple Regression Models of the Prospective Associations Between Children’s Mean Self-Reported Perceived Racial Discrimination Score and Measures of Adiposity^a^

Variable	Participant, β (95% CI) (N = 6463)			
	Year 2 BMI *z *score		Year 2 waist circumference	
	Unadjusted	Adjusted	Unadjusted	Adjusted
Perceived racial discrimination	0.05 (0.02 to 0.08)^b^	0.04 (0.01 to 0.08)^c^	0.35 (0.15 to 0.54)^b^	0.24 (0.04 to 0.44)^c^
Age, y	NA	0.01 (−0.01 to 0.03)	NA	0.21 (0.09 to 0.32)^b^
Sex				
Male	NA	1 [Reference]	NA	1 [Reference]
Female	NA	0.09 (0.07 to 0.12)^b^	NA	−0.04 (−0.18 to 0.10)
Nativity status				
US born	NA	1 [Reference]	NA	1 [Reference]
Non-US born	NA	0.01 (−0.06 to 0.09)	NA	−0.44 (−0.86 to −0.01)^c^
Total household income, $				
24 999 or less	NA	1 [Reference]	NA	1 [Reference]
25 000-49 999	NA	−0.05 (−0.10 to 0.00)^d^	NA	−0.12 (−0.41 to 0.16)
50 000-74 999	NA	−0.07 (−0.12 to −0.01)^c^	NA	−0.22 (−0.52 to 0.07)
75 000-99 999	NA	−0.08 (−0.13 to −0.02)^b^	NA	−0.30 (−0.60 to −0.00)^c^
100 000-199 999	NA	−0.09 (−0.14 to −0.04)^b^	NA	−0.42 (−0.70 to −0.14)^b^
200 000 and above	NA	−0.10 (−0.16 to −0.04)^b^	NA	−0.44 (−0.78 to −0.11)^b^
Parent education level				
High school diploma or less	NA	1 [Reference]	NA	1 [Reference]
Some college	NA	0.04 (−0.01 to 0.09)	NA	−0.14 (−0.42 to 0.13)
Associate’s or Bachelor’s	NA	0.01 (−0.03 to 0.06)	NA	−0.15 (−0.40 to 0.11)
Graduate degree	NA	−0.01 (−0.06 to 0.04)	NA	−0.45 (−0.74 to −0.17)^b^
Year 1 BMI *z *score	0.89 (0.88 to 0.90)^b^	0.89 (0.87 to 0.90)^b^	NA	NA
Year 1 waist circumference, in	NA	NA	0.86 (0.84 to 0.87)^b^	0.84 (0.83 to 0.86)^b^
Constant	0.06 (0.05 to 0.07)^b^	0.00 (−0.20 to 0.20)	4.93 (4.50 to 5.35)^b^	3.78 (2.59 to 4.96)^b^
Observations, No.	6463	6463	6463	6463
*R^2^*	0.81	0.81	0.66	0.66

Abbreviations: BMI, body mass index (calculated as weight in kilograms divided by height in meters squared); NA, not applicable.

^a^ Age- and sex-adjusted BMI *z *score and waisted circumference.

^b^ *P* <.01.

^c^ *P* <.05.

^d^ *P* <.1.

## Discussion

We examined the prospective association between racial discrimination and adiposity (BMI *z *score and waist circumference) 1 year later in a nationally representative sample of 9- to 11-year-olds. Even after adjusting for covariates (ie, age, sex, household income, parent education, and nativity status), racial discrimination was positively associated with both indicators of youth adiposity.

Racial discrimination is a social determinant of a wide range of chronic conditions in children and adolescents.^14,20,21,42^ We found that racial discrimination was positively associated with adiposity over time. Racial discrimination, as a psychosocial stressor, can lead to a higher BMI through biobehavioral pathways. While the mechanisms through which discrimination increases BMI and waist circumference among youths are not yet fully understood, studies have shown that exposure to discrimination can lead to changes in cortisol levels, such as flattened diurnal cortisol slopes and reduced cortisol awakening responses,^21^ as well as unhealthy eating habits,^43^ sleep problems,^16^ and poor mental health^20,21^ in children and adolescents. To date, there is a lack of research examining the potential mediating factors in the association between racial discrimination and adiposity in children and adolescents. The ABCD study provides an opportunity to leverage multiple data sets, including measures of diet and brain imaging, to gain a better understanding of the association between discrimination and adiposity in this study sample. Further studies should aim to explore the biobehavioral pathways (eg, physical activity,^44^ screen time,^44^ nucleus accumbens^46^) that may help to explain this association in children and adolescents.

Examining intersecting identities with racial discrimination is an important area of focus in obesity research because exposure to racial discrimination can be influenced by other identities, such as gender, sexual orientation, socioeconomic status, and more. For instance, Black children from high-income families experience higher levels of racial discrimination compared with Black children from lower-income familes.^34^ This aligns with the existing literature, which indicates that Black US residents with higher socioeconomic status tend to report more discrimination than Black children with a lower socioeconomic status.^37^ In addition, studies suggest that Black boys report more racial discrimination than Black girls.^47,48^ Thus, it is important to recognize the complex interplay of these identities because they may have differential influence in the association between discrimination and health outcomes. Further research with a larger sample size is needed to explore how intersectional identities, such as Black boys with a high socioeconomic status, may intersect with discrimination exposure to increase the risk of high BMI. By understanding how intersecting identities relate to discrimination experiences and health outcomes, we can develop more targeted and effective interventions to reduce health disparities.

### Limitations

This study has limitations. The analysis in this study was limited to complete cases, which may result in selection bias, as participants with missing data on the variables of interest may differ from those with complete data in ways that are related to experiences of discrimination and BMI levels. Consequently, the associations observed in this study may not be generalizable to the broader population. We used 2 measures of adiposity. While BMI *z *score and waist circumference are reliable and valid measures for youth adiposity,^49^ other adiposity measures may have stronger efficiency in identifying clinical risks in children and adolescents.^50^ In future studies, use of multiple adiposity measures, such as body fat percentage or subcutaneous fat, will advance understanding of the association of racial discrimination on overweight and obesity risk.

While the estimate of a 0.04 mean higher BMI *z *score and 0.24-inch waist circumference with each increase in mean discrimination score may suggest a small statistical association, it is important to recognize that discrimination may not have an immediate strong association with adiposity. However, prolonged exposure to racial discrimination, in combination with other forms of racism, may strengthen the association between racial discrimination and adiposity over time. Therefore, even repeated small experiences of discrimination could impact risk of obesity, especially for those who are already vulnerable. This study lays the groundwork for further research to explore the longitudinal relationship between discrimination and BMI, including the potential impact of cumulative exposure on the development of obesity.

Understanding the associations of discrimination on adiposity across life course stages, especially in early childhood, is needed to develop effective and targeted prevention strategies at critical development points. In this study, only 1 type of discrimination was examined, specifically discrimination based on race or ethnicity. However, other forms of discrimination, such as sexism, weight bias, and heterosexism, are also social determinants of health and health disparities. The focus on only 1 form of discrimination does not provide a complete understanding of how discrimination impacts BMI. Furthermore, the reported racial discrimination experienced by White youths raises questions about their interpretation of the measure items compared with youth from racially minoritized groups. Further research is needed to comprehend how youths construct meaning from negative experiences that may be perceived as discriminatory. Additionally, the study’s racial discrimination scale only asked about discrimination from teachers, peers, and other adults, which may not capture all forms of discrimination experienced by participants. Therefore, future studies using discrimination measures that encompass the full range of experiences is critical to comprehending the association of discrimination with BMI.

## Conclusions

Racial discrimination has adverse effects on a range of health outcomes.^20,21,42^ We found that racial discrimination exposure was positively and significantly associated with BMI and waist circumference over time, even when adjusting for known socioeconomic risk factors for excess adiposity, such as income. Our study highlights the need for a multifaceted approach to address racial discrimination and its impacts on the health of children and adolescents. Researchers, clinicians, educators, and policy makers should collaborate with communities to implement evidence-based strategies to prevent racial discrimination and improve the health outcomes of those disproportionately affected. One potential approach could be to optimize clinical care among pediatricians by screening patients who report experiencing racism for mental health conditions and integrating positive youth development approaches that focus on identifying strengths and protective factors.^51^ Additionally, there is a need for the development of theory-driven policies, practices, and curricula that address personally mediated racial discrimination and that are developed through community-based methods with a focus on prevention.^51^ Incorporating elements from Aaron and Standford’s 10-point strategy^52^ can be effective, including reducing economic inequality, reinforcing the social safety net, and strengthening nondiscrimination protections. Addressing racial discrimination is crucial to prevent longstanding racial obesity disparities and improve the health and life outcomes of future generations.^53,54,55^

## Notes

Supplement 1. **eAppendix.** Perceived Discrimination Scale

Supplement 2.
Data Sharing Statement
