# Supplementary material for: Association of Racial Discrimination With Adiposity in Children and Adolescents
Source: JAMA Netw Open. 2023 Jul 11;6(7):e2322839. doi: 10.1001/jamanetworkopen.2023.22839 (PMC10336613; doi:10.1001/jamanetworkopen.2023.22839)
Supplement: Supplement 1. — eAppendix. Perceived Discrimination Scale [file jamanetwopen-e2322839-s001.pdf]

Supplemental Online Content

Cuevas AG, Krobath DM, Rhodes-Bratton B, et al. Association of racial discrimination with adiposity in children and adolescents. *JAMA Netw Open*. 2023;6(7):e2322839. doi:10.1001/jamanetworkopen.2023.22839

eAppendix. Perceived Discrimination Scale

This supplemental material has been provided by the authors to give readers additional information about their work.

© 2023 Cuevas AG et al. *JAMA Network Open.*

eAppendix.

Perceived Discrimination Scale

Reference: Phinney JS, Madden T, Santos LJ. Psychological Variables as Predictors of Perceived Ethnic Discrimination Among Minority and Immigrant Adolescents. *Journal of Applied Social Psychology*. 1998;28(11):937-953.

Based on a 5-point Likert scale, as follows: 0 (almost never), 1 (seldom), 2 (sometimes), 3 (often), and 4 (very often)

How often do the following people treat you unfairly or negatively because of your ethnic background?

1. Teachers

2. Other adults outside school

3. Other students

4. I feel that others behave in an unfair or negative way toward my ethnic group.

Because of my ethnic background:

5. I feel that I am not wanted in American society.

6. I don't feel accepted by other Americans.

7. I feel that other Americans have something against me

© 2023 [First author last name and initials or group name] et al. *[Journal name].*
