# Supplementary material for: Association of Racial Discrimination With Adiposity in Children and Adolescents
Source: JAMA Netw Open. 2023 Jul 11;6(7):e2322839. doi: 10.1001/jamanetworkopen.2023.22839 (PMC10336613; doi:10.1001/jamanetworkopen.2023.22839)
Supplement: Supplement 2. — Data Sharing Statement [file jamanetwopen-e2322839-s002.pdf]

Data Sharing Statement

Cuevas. Association of Racial Discrimination With Adiposity in Children and Adolescents. *JAMA Netw Open*. Published July 11, 2023. doi:10.1001/jamanetworkopen.2023.22839

Data
Data available: No

Additional Information
Explanation for why data not available: Data described in the article, code book, and analytic code will be made available upon request.
